# Uganda public health fellowship program’s contribution to building a resilient and sustainable public health system in Uganda

**DOI:** 10.1080/16549716.2019.1609825

**Published:** 2019-05-23

**Authors:** Alex Riolexus Ario, Lilian Bulage, Daniel Kadobera, Benon Kwesiga, Steven N. Kabwama, Patrick Tusiime, Rhoda K. Wanyenze

**Affiliations:** a Ministry of Health of Uganda, Kampala, Uganda; b Uganda National Institute of Public Health, Kampala, Uganda; c Uganda Public Health Fellowship Program, Ministry of Health, Kampala, Uganda; d African Field Epidemiology Network, Kampala, Uganda; e Uganda Public Health Fellowship Program and Makerere University School of Public Health, Kampala, Uganda

**Keywords:** Field epidemiology, resilience, sustainability, health systems, Uganda

## Abstract

**Background**: Low-income countries with relatively weak-health systems are highly vulnerable to public health threats. Effective public health system with a workforce to investigate outbreaks can reduce disease impact on livelihoods and economic development. Building effective public health partnerships is critical for sustainability of such a system. Uganda has made significant progress in responding to emergencies during the past quarter century, but its public health workforce is still inadequate in number and competency.

**Objectives**: To reinforce implementation of priority public health programs in Uganda and cultivate core capacities for compliance with International Health Regulations.

**Methods**: To develop a competent workforce to manage epidemics and improve disease surveillance, Uganda Ministry of Health (MoH) established an advanced-level Field Epidemiology Training Program, called Public Health Fellowship Program (PHFP); closely modelled after the US CDC’s Epidemic Intelligence Service. PHFP is a 2-year, full-time, non-degree granting program targeting mid-career public health professionals. Fellows spend 85% of their field time in MoH placements learning through service delivery and gaining competencies in major domains.

**Results**: During 2015–2018, PHFP enrolled 41 fellows, and graduated 30. Fellows were placed in 19 priority areas at MoH and completed 235 projects (91 outbreaks, 12 refugee assessments, 50 surveillance, and 60 epidemiologic studies, 3 cost analysis and 18 quality improvement); made 194 conference presentations; prepared 63 manuscripts for peer-reviewed publications (27 published as of December 2018); produced MoH bulletins, and developed three case studies. Projects have resulted in public health interventions with improvements in surveillance systems and disease control.

**Conclusion**: During the 4 years of existence, PHFP has contributed greatly to improving real-time disease surveillance and outbreak response core capacities. Enhanced focus on evidence-based targeted approaches has increased effectiveness in outbreak response and control, and integration of PHFP within MoH has contributed to building a resilient and sustainable health system in Uganda.

## Background

The burden of communicable and non-communicable diseases (NCDs) in Uganda is high. Most major health outcome indicators fall well short of desired targets: 343 women die for every 100,000 live births, and 131 of every 1,000 children die before age 5 years. Nearly one in three children under 5 years is stunted [1]. More than half of the disability-adjusted life-years lost in Uganda are due to communicable diseases, in part due to the high HIV prevalence (6.2%) and TB prevalence (3/1000) [2]. The emergence of multidrug-resistant tuberculosis has spread countrywide, and the growing non-communicable disease burden is of increasing concern [3,4] As an ecological hotspot, Uganda has infectious disease transmission belts for meningitis, yellow fever, and viral haemorrhagic fevers. The country is prone to emerging and re-emerging infectious diseases, most of which have occurred in epidemic proportions in recent times with significant cost implications. For example, the cost of responding and controlling the 2017 Marburg Virus Disease outbreak in Kween District, Eastern Uganda was approximately $1 million USD [5]. Addressing all of these health challenges requires a resilient health system, if meaningful prevention and control is to be achieved.

**Figure 1.**
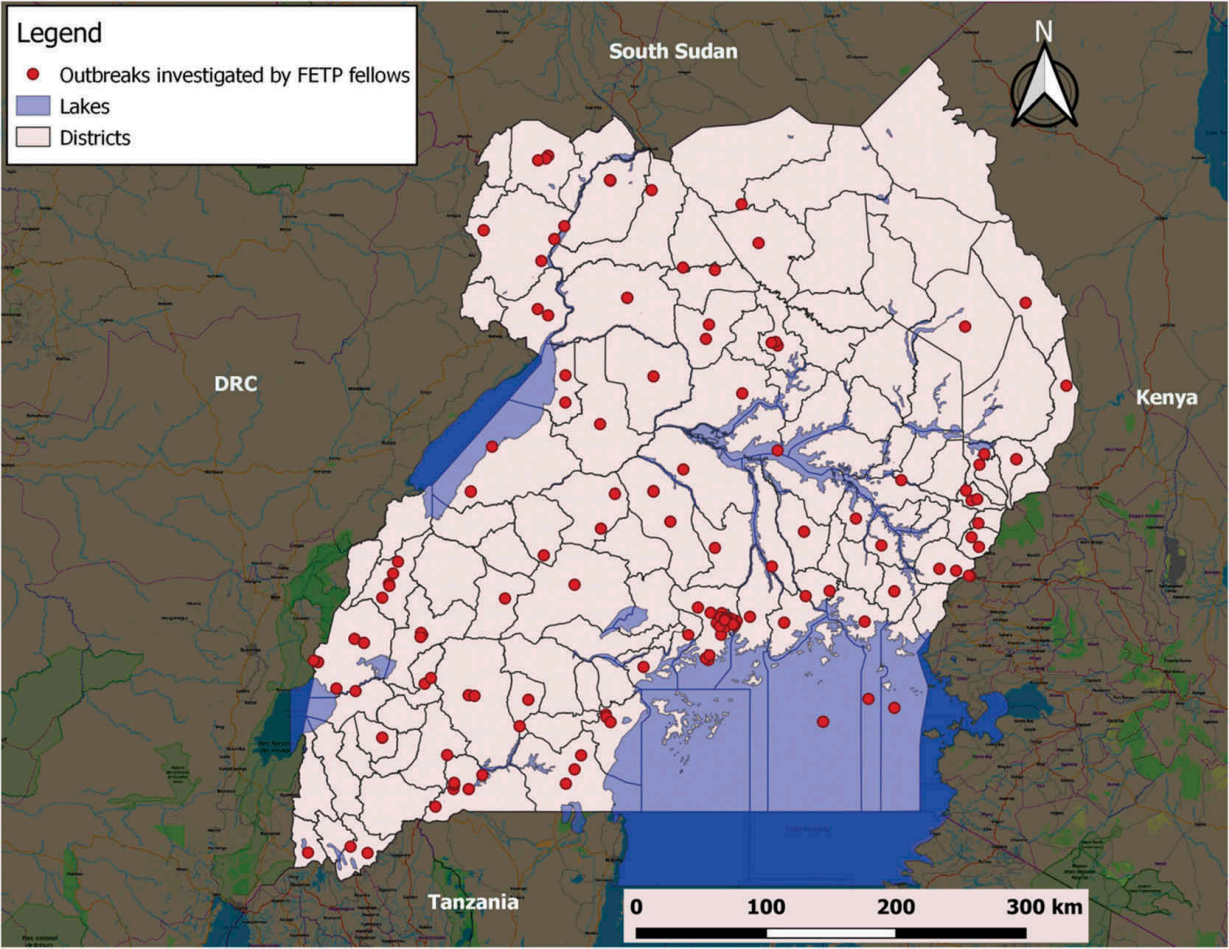
Map of Uganda showing distribution of outbreaks investigated by FETP Fellows countrywide, 2015–2018.

Health system resilience comprises both health system strengthening and sustainability. Health system strengthening refers to significant and purposeful efforts to improve the system’s performance, while sustainability has been defined as the implementation and continuous use of new practices that are able to produce the intended outcomes over a long period of time [6]. The World Health Organisation (WHO) in 2007 developed a framework that describes health system strengthening in terms of six building blocks: service delivery, health workforce, health information systems, access to essential medicines, financing, and leadership/governance [7].

Health workforce challenges have been recognized as a critical bottleneck to the delivery of high-quality health services, including response to epidemics. The spread of Ebola in West Africa during 2013–2016 was compounded by weak-health systems characterized by lack of public health capacity for outbreak detection and control [8,9]. While there is no doubt that new strategies are needed to respond to evolving systems challenges, even the current available human resources are not being used to sufficiently strengthen the performance of health systems. Evidence-informed policy-making presupposes the availability of high quality, relevant information, and decision-makers may need support to assess what is already known or to articulate demands for specific new evidence.

Field epidemiologists respond to public health emergencies, including outbreaks, as well as conducting epidemiologic research, evaluating and improving surveillance systems, implementing public health programs, and publishing data to facilitate evidence-based decision-making. The shortage of field epidemiologists in Uganda to address critical aspects of health in the public sector prompted the Uganda Ministry of Health (MoH), with support of key partners including Makerere University School of Public Health (MakSPH) and US Centers for Disease Control and Prevention (CDC), to establish the Uganda Public Health Fellowship Program (PHFP) in 2015. PHFP is an in-service, post-master’s-degree field epidemiology training program (FETP) that attempts to address human resources for health needs. As part of the MoH’s long-term sustainability vision, PHFP will exist as a directorate and capacity-building arm of the Uganda National Institute of Public Health (UNIPH); UNIPH is an initiative of the MoH to create an integrated disease control centre, analogous to the US CDC. This paper describes the PHFP, its development and organization, and its contribution to building a resilient and sustainable health system by training a critical mass of competent field epidemiologists in Uganda.

## Methods

### Program description

The PHFP is a 2-year, non-degree-granting, full-time, competency-based fellowship program modelled after the US CDC’s Epidemic Intelligence Service (EIS) program. The program is primarily funded by the US Government through the President’s Emergency Plan for AIDS Relief (PEPFAR), the President’s Malaria Initiative (PMI), and the Global Health Security Agenda (GHSA). The PHFP trains mid-career professionals who have a master’s degree (or higher) in a health-related discipline and who aspire to become public health leaders. During the two-year fellowship period, fellows are required to attain certain core competencies in domains that include public health emergency response, surveillance data analysis, surveillance system evaluation, applied epidemiologic study, cost analysis of outbreaks, quality improvement science, burden of disease estimation, and leadership skills. Their attainment of these competencies is demonstrated by completing a portfolio of projects in each of these domains.

PHFP is integrated as an arm of MoH, and, together with the Integrated Epidemiology and Surveillance Department (IESD), the Public Health Emergency Operations Centre (PHEOC), and other important public health programs in the MoH, conducts investigations and studies that provide data for decision-making for the National Task Force (NTF). The NTF is an arm of the MoH created to coordinate emergency health response, and is responsible for bringing partners together, providing strategic direction to response, and coordinating functions that contribute to prevention and control of public health emergencies.

The PHFP Secretariat is the operational and administrative unit responsible for coordinating the program. Its functions include: (a) coordination of PHFP partner collaboration with MoH, (b) organizing and supervising fellows’ placements within MoH, (c) performance review of fellows, (d) back-up mentorship for Fellows and coordination of mentorship activities, and (e) development of strategic and operational plans for approval by the PHFP Steering Committee (SC). The PHFP secretariat comprises a field coordinator, two field supervisors, a scientific writer, a training manager, and an administrative assistant, based at the Ministry of Health. The CDC Resident Advisor, who provides technical and programmatic guidance, supports the Secretariat, but is not a part of it.

### Recruitment and training

Applications are solicited through the print media, the program website, the alumni association, and professional associations. Short-listed candidates are required to undergo a rigorous interview process comprising an in-person interview, a Power point presentation on a given relevant topic, and an essay on a selected topic. Approximately 10 fellows are selected annually.

The service-in-training program comprises two didactic courses of approximately six weeks in duration each (~15% of the program time): an introductory course at the beginning, and an advanced course towards the end of the first year. The remaining weeks (~85% of time), the fellows are placed at an MoH host site, where they implement activities stipulated in their scope of work and work plans, tailored towards accomplishment of host site activities and fellowship deliverables. The hands-on-learning is anchored around intense continuous mentorship by mentors at the host site, the Secretariat, and staff at Makerere University College of Health Sciences.

Fellows’ progress is monitored and performance appraised on a quarterly and annual basis. Supportive supervision is offered to the host site to ensure prompt identification of problems and possible solutions.

## Results

***Enrolment***: The program was launched in January 2015 with 10 fellows in the first cohort. In 2018, the 4th cohort was enrolled, and in August 2018, the 5th cohort was selected. The four cohorts of 41 fellows (10 each in Cohorts 2015, 2016 and 2018, and 11 in Cohort 2017) were recruited and trained with each group commencing in January of the year of enrolment; the 5th cohort of 14 fellows commenced their fellowship in January 2019. Fellows entering the program held various advanced degrees in general public health (MPH or MScPH, 22 fellows), medicine (MMed, 1 fellow), epidemiology or clinical epidemiology (MSc, 8 fellows), entomology/parasitology/zoology (MSc, 2 fellows), international health/public health (MSc, 2 fellows), and health services research/management (MSc, 2 fellows). One fellow each have also held degrees in veterinary preventive medicine (VPM), medical microbiology (MSc), infectious disease management (MSc), and health policy (MSc). Applications increased from 44 in 2015 to 195 in 2017, but dropped to 83 and 89 in 2018 and 2019. Fellows have been placed in 19 MoH priority areas during their training (Table 1).

**Table 1.** Host sites for fellows of the Uganda Public Health Fellowship Program, 2015–2018.

Placement site	Number of fellows
**Ministry of Health**	
Epidemiology and Surveillance Division	2
National Malaria Control Program	4
STD/AIDS Control Program	3
Division of Health Information	3
Uganda National Expanded Program on Immunization	4
Mental Health and Substance Abuse	3
National Health Laboratory Services	2
National Tuberculosis and Leprosy Program	3
Reproductive Health Division	3
Neglected Tropical Diseases Program	2
Emergency Operations Centre/Anti-microbial Resistance	1
**Ministry of Health-Affiliated Institutions**	
Mildmay Uganda	1
Uganda Virus Research Institute	2
Uganda Cancer Institute	2
National Animal Disease Epidemiology and Diagnostics Centre	2
Uganda Prisons Health Services	1
**District/City Health Office**	
Tororo District Health Office/BASIIN Study	1
Rakai District Health Office/Rakai Health Sciences Program	1
Kampala Capital City Authority	1

***Graduation***: By January 2019, the first three cohorts of fellows had graduated. All graduates have been absorbed within the MoH (53%) or are working with MoH-affiliated or linked institutions or organisations [WHO, IDI, AFENET and Africa CDC] (47%) (Table 2).

**Table 2.** Absorption of graduates.

Year of Graduation	No	Institution/Program	No	%
2017	10	Uganda Public Health Fellowship Program˜	3	30%
		African Field Epidemiology Network	2	20%
		Infectious Diseases Institute	1	10%
		National Malaria Control Program˜	1	10%
		AIDS Control Program˜	1	10%
		Vector Control Division˜	1	10%
		World Health Organization	1	10%
2018	9	National Tuberculosis & Leprosy Program˜	1	11%
		Infectious Diseases Institute	1	11%
		Makerere University School of Public Health	2	22%
		Africa CDC	1	11%
		Joint Clinical Research Centre	1	11%
		Epidemiology and Surveillance Division˜	1	11%
		Division of Health Information˜	1	11%
		Mildmay Uganda	1	11%

˜Ministry of Health Department/Program

***Field projects***: During the nearly four years of the program implementation, the 41 PHFP fellows completed more than 235 applied epidemiology projects, of which 91 were outbreak investigations and 12 were emergency refugee health assessments. In addition, fellows have conducted 50 surveillance projects, including analysis of surveillance data or evaluation of a surveillance system, and completed 60 epidemiological studies, 18 quality improvement projects, and 3 cost analyses for outbreaks (Table 3). On average, each Fellow implemented 8 projects. These field investigations have been conducted throughout the country (Figure 1).

**Table 3.** Achievements by the fellows of the Uganda Public Health Fellowship Program, 2015–2018.

	2015	2016	2017	2018	Total
**Field projects participated**	**33**	**62**	**59**	**81**	**235**
Outbreak investigations	18	15	27	31	91
Emergency investigations	1	4	7	0	12
Surveillance	9	16	15	10	50
Applied epidemiologic studies	5	22	5	29	61
Cost analysis of outbreaks	0	2	1	0	3
Quality Improvement	0	3	4	11	18
**Scientific publications co-authored**	**9**	**48**	**87**	**51**	**195**
Peer-reviewed, published or accepted	0	0	7	20	27
Peer-reviewed, submitted, not published yet	1	10	17	9	37
MoH Quarterly Epi Bulletin articles	8	38	63	22	131
**Presentations made at scientific conferences**	**23**	**47**	**61**	**63**	**194**
International conferences	3	16	25	36	80
National conferences	20	31	36	27	114
**Awards received**	**0**	**2**	**2**	**1**	**5**

***Communication outputs***: PHFP has submitted 63 manuscripts for publication in peer-reviewed journals; 25 were published as of December 2018. A total of 29 manuscripts are undergoing internal and CDC reviews. Findings from some of the published manuscripts, such as Risk Factors for Podoconiosis in Kamwenge District, Uganda [10] were widely covered in major national and international media outlets, including international media such as CNN and the New York Times [11,12] In addition to the manuscripts, PHFP has had 194 abstracts accepted for presentation at national and international conferences. The program also supported the MoH in revamping three epidemiology bulletins and developing three new bulletins. These three bulletins (*Neglected Tropical Diseases Bulletin, National TB and Leprosy Program Bulletin*, and *Non-Communicable Diseases Bulletin*) serve as sites where the fellows and other MoH epidemiologists publish valuable public health information for immediate local use. The quarterly MoH Epidemiological Bulletin has published 131 articles based on the fellows’ projects, and the Weekly Epidemiological Bulletin has been running since August 2015 with fellows taking a lead in its production. National newspapers have also published articles from many fellows, written to inform the public about current public health challenges, such as outbreaks and tips on disease prevention. These publications have contributed to use of their public health recommendations on a wider scale nationally.

***Contribution to building a resilient system***: Prior to PHFP, there was no investigative arm for outbreak response at MoH; the PHFP created and now leads this arm, as part of the National Rapid Response Team. Outbreak reports prepared by fellows are routinely submitted to the PHEOC and presented at the National Task Force for Epidemic Preparedness and Response, which helps to guide outbreak prevention and control in the country. Examples include

identification and investigation of a large typhoid outbreak in 2015, which affected over 10,000 residents in Kampala, in which investigation findings helped to guide the successful control of the outbreak, early identification and investigation of a yellow fever outbreak in 2016, leading to a subsequent mass vaccination campaign by MoH, and investigation of a meningitis (serogroup W) outbreak in an institutionalized population in 2016, with subsequent control efforts potentially preventing spread into the civilian population. PHFP also provided epidemiologic support for nationwide outbreaks of cholera, malaria, measles and anthrax [13]. PHFP fellows have also conducted emergency assessments and evaluations at several refugee settlement areas. Implementation of recommendations from these investigations by MoH and UN agencies has potentially prevented outbreaks and improved the health of the refugees.

The prompt epidemiologic investigations conducted by the PHFP have resulted in shortened time to identify dangerous pathogens, and prevented potential spread of outbreaks. For example, due to timely investigation and prompt response, a yellow fever outbreak that occurred in 2016 was confirmed within 12 days and controlled within three weeks of the initial outbreak report. In comparison, the previous yellow fever outbreak in Uganda in 2010 took 40 days to confirm and 3 months to control [14]. Due to its achievements, PHFP won the CDC Director’s Award for Excellence in Public Health and Response at the 2017 EIS conference [15].

PHFP and the MoH are also supported by a Frontline FETP program, established by the MoH in partnership with the CDC in 2016. Frontline FETP augments disease surveillance and outbreak detection and response at the district level [16].

## Discussion

In just four years of operation, Uganda’s Public Health Fellowship Program has demonstrated the ability to address multiple gaps in the Uganda health system. They have generated quality products, experienced adoption of recommendations from their projects, and received national and international recognition [17]. As an integrated and adaptive program, PHFP has greatly improved Uganda’s capacity to respond to disease outbreaks and other public health emergencies, controlling them at their sources, in a timely and effective manner.

Much as established literature guides response to epidemics, we are aware that each outbreak is unique. Before PHFP, outbreak responses in Uganda typically had only minimal epidemiological investigation [16]. The commencement of PHFP brought systematic approaches to epidemiologic investigations in almost all outbreaks in Uganda [18–20]. As a result, PHFP has made the National Rapid Response Team more focused and enabled the MoH to control outbreaks in a shorter time period and at lower cost. The decisions taken by the National Task Force are now routinely informed by evidence generated by PHFP.

Vertical MoH programs have benefitted from having embedded Fellows routinely analyse their data and ensure that alerts are generated promptly. Because PHFP is non-degree awarding and Fellows are required to already have at least a Master’s degree on entry, fellows spend more time working on program-oriented projects that address national health needs, and less time in didactic courses. This is different from most FETPs, in which residents earn degrees while in their programs [21]. This model allows the fellows to obtain wide range of experience at all levels of public health in Uganda. Fellows have also demonstrated this capability by producing more manuscripts and presenting a number of papers at national and international level within a short span of time. Compared to the evaluation report of the FETP at Chennai, Tamil Nadu, India whose fieldwork led to the production of 158 scientific communications presented at international meetings and to 29 manuscripts accepted in indexed, peer-reviewed journals in seven years. Going by this trend, achievement of PHFP will definitely be greater than most FETPs [22].

Beyond the health benefits to the country, PHFP and similar programs almost certainly provide return on investment through early detection, investigation and control of outbreaks, improvement in surveillance system, and provision of urgently needed data for public health programs. The recent Ebola outbreak in West Africa cost the global community $3.6B to respond to and contain and an additional $2.2B in GDP loss to Guinea, Liberia, and Sierra Leone [23]. Had a fraction of that sum of money been used to build an effective health workforce in West Africa for early identification, investigation, and control of outbreaks, both the human and the massive economic toll of the Ebola epidemic could perhaps have been averted. The importance of putting people at the centre of delivery of health services was apparent during the initial response, the early recovery phase, and long-term planning for resilience in the Ebola response [24]. Other recent outbreaks, including the 2012 Middle East Respiratory Syndrome coronavirus and the 2015 Zika virus outbreaks, have similarly underscored the need for strong, resilient public health systems to both address the outbreaks and implement containment measures.

All fellows who have graduated have been absorbed either within MoH or are employed by institutions that work closely with the MoH. Because the program Secretariat and most host sites are in the MoH, having graduates working in the different programs within the MoH provides opportunity for mentorship of new fellows (Table 2). Increased retention of graduates within the Ugandan government will facilitate sustainability of the program. Presently, recruitment in Ministry of Health is the prerogative of the Health Service Commission which derives its mandate from Public Service Regulations. These regulations in its current form does not ring fence any positions for FETP graduates, however, discussion are on the table by all relevant stakeholders to incorporate a clause that will make it easier to recruit and retain FETP graduates in public service. It’s important to note that Government of Uganda (GoU) intends to have 3300 field epidemiologists trained to match the current population based on the WHO target of 1 epidemiologist per 200,000 population. Based on the recruitment of 10 fellows per year and the current reliance on donor funding, it will definitely take a relatively long time to bridge this gap. However, GoU’s plan to have a fully functional UNIPH which is largely funded by government in the very near future will definitely help in addressing this challenge. In addition, Makerere School of Public Health has produced 340 FETP graduates since inception in 1994.

Although PHFP has made strides in contributing to building a resilient system, there are still some challenges. The use of evidence generated for disease prevention and control is still limited, partially due to the multiple layers of implementation existing in the Ugandan system. Even when evidence is available, poor dissemination, rigid mindset, poor coordination of partners, and inadequate resources may hamper its utilization. Improved stakeholder engagement with the MOH should be able to address this challenge in the long run. Funding also has some challenges: although PHFP is currently funded by the USA Government, for full integration and institutionalization within the Uganda MoH system there must be domestic resource allocation to support this program. Recognizing this need, MoH has made PHFP a key component of the proposed Uganda National Institute of Public Health (UNIPH) by designating it as a unique directorate [25]. Once the UNIPH is formally established by the Ugandan Parliament, it will become an integrated disease control centre in the country and have diversified funding sources from the government, philanthropists, and the private sector, as well as grants and cooperative agreements from international organisations and foreign governments. PHFP is intended to be its capacity-building component, which will provide a competent workforce of field epidemiologists and other health professionals to meet the public health needs of the country [26]. Moreover, PHFP alumni have formed an association called ‘Field Epidemiologists without Borders’, which will work closely with UNIPH to champion some of the institute’s objectives to ensure knowledge transfer and the building of a critical mass of field epidemiologists [27].

## Conclusion

During its four years of operation, the PHFP has contributed greatly to improving the real-time disease surveillance and outbreak response core capacities of the Uganda Ministry of Health. The enhanced focus on evidence-based targeted approaches has increased effectiveness in outbreak response and control, and the integration of PHFP within the MoH has contributed to building a resilient and sustainable health system in Uganda.

## Paper context

PHFP has contributed greatly to improving the real-time disease surveillance and outbreak response core capacities of the Uganda Ministry of Health within a 4-year span. The enhanced focus on evidence-based targeted approaches has increased effectiveness in outbreak response and control, and the integration of PHFP within the MoH has contributed to building a resilient and sustainable health system in Uganda.
